# Supplementary material for: The longevity effects of reduced IGF-1 signaling depend on the stability of the mitochondrial genome
Source: bioRxiv. 2025 Jun 6:2025.06.03.656903. Preprint. [Version 1] doi: 10.1101/2025.06.03.656903 (PMC12157428; doi:10.1101/2025.06.03.656903)
Supplement: 3 [file NIHPP2025.06.03.656903v1-supplement-3.pdf]

bioRxiv preprint doi: https://doi.org/10.1101/2025.06.03.656903; this version posted June 6, 2025. The copyright holder for this preprint (which was not certified by peer review) is the author/funder, who has granted bioRxiv a license to display the preprint in perpetuity. It is made available under aCC-BY 4.0 International license.

Figure S1

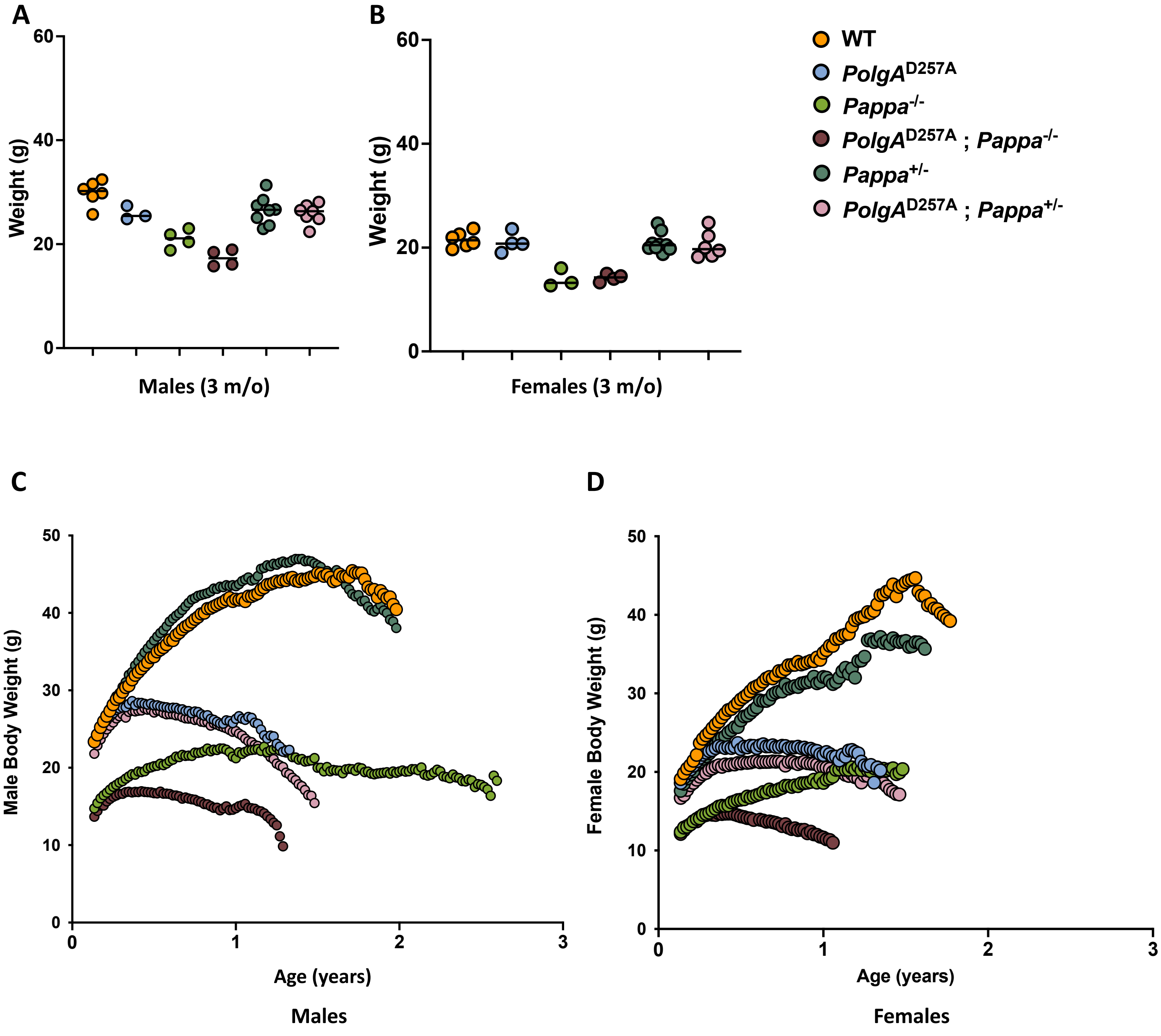

Figure S1| Weight distribution of young WT and mutant mice, and progression throughout their lifespan. A. Weight distribution of 3-month-old male mice (n=3-9/group). **B.** Weight distribution of 3-month-old female mice (n = 4-9/group). **C.** Weight distribution of male mice throughout their lifespan. **D.** Weight distribution of female mice throughout their lifespan.

bioRxiv preprint doi: https://doi.org/10.1101/2025.06.03.656903; this version posted June 6, 2025. The copyright holder for this preprint (which was not certified by peer review) is the author/funder, who has granted bioRxiv a license to display the preprint in perpetuity. It is made available under aCC-BY 4.0 International license.

Figure S2

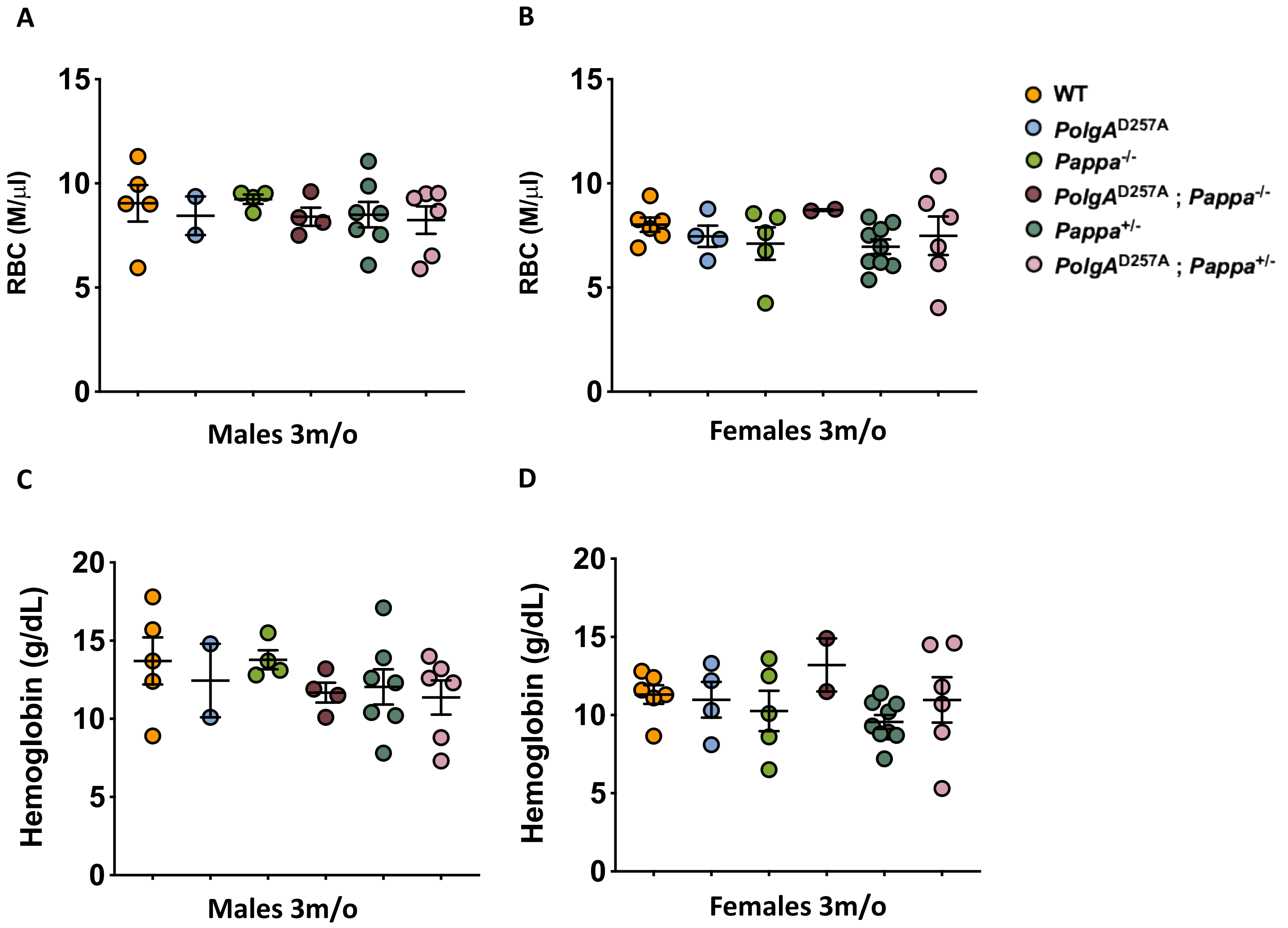

Figure S2| Red blood cell count and hemoglobin content in young WT and mutant mice. A. RBC count in 3-month-old male mice. **B.** RBC count in 3-month-old female mice. **C.** Hemoglobin content in 3-month-old male mice. **D.** Hemoglobin content in 3-month-old female mice. Male n = 2-7/group, female n = 2-9/group.

bioRxiv preprint doi: https://doi.org/10.1101/2025.06.03.656903; this version posted June 6, 2025. The copyright holder for this preprint (which was not certified by peer review) is the author/funder, who has granted bioRxiv a license to display the preprint in perpetuity. It is made available under aCC-BY 4.0 International license.

Figure S3

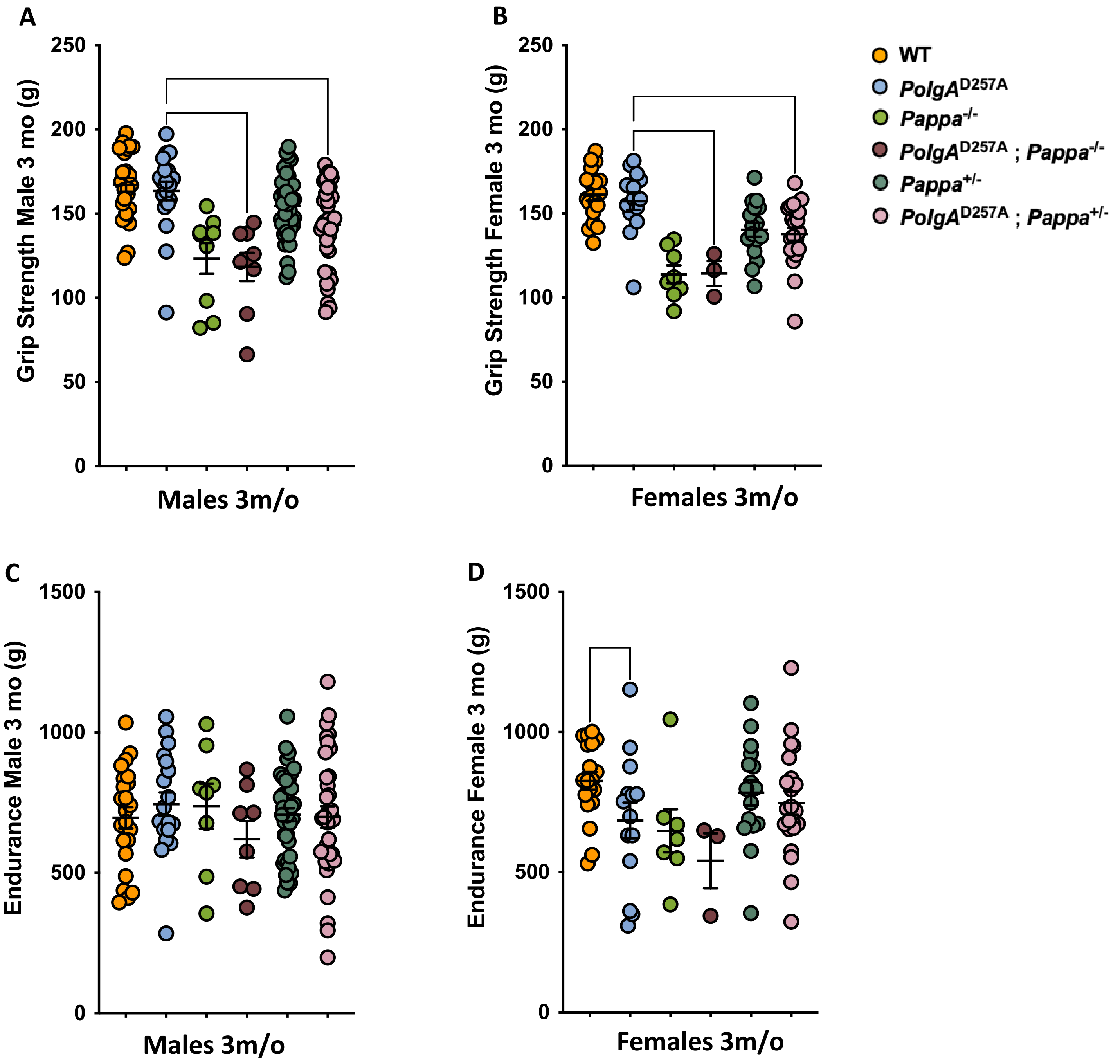

Figure S3| Grip strength and endurance in young WT and mutant mice. A. Grip strength in 3-month-old male mice. **B.** Grip strength in 3-month-old female mice. **C.** Endurance in 3-month-old male mice. **D.** Endurance in 3-month-old female mice. Male n = 9-40/group, female n = 3-22/group).

bioRxiv preprint doi: https://doi.org/10.1101/2025.06.03.656903; this version posted June 6, 2025. The copyright holder for this preprint (which was not certified by peer review) is the author/funder, who has granted bioRxiv a license to display the preprint in perpetuity. It is made available under aCC-BY 4.0 International license.

Figure S4

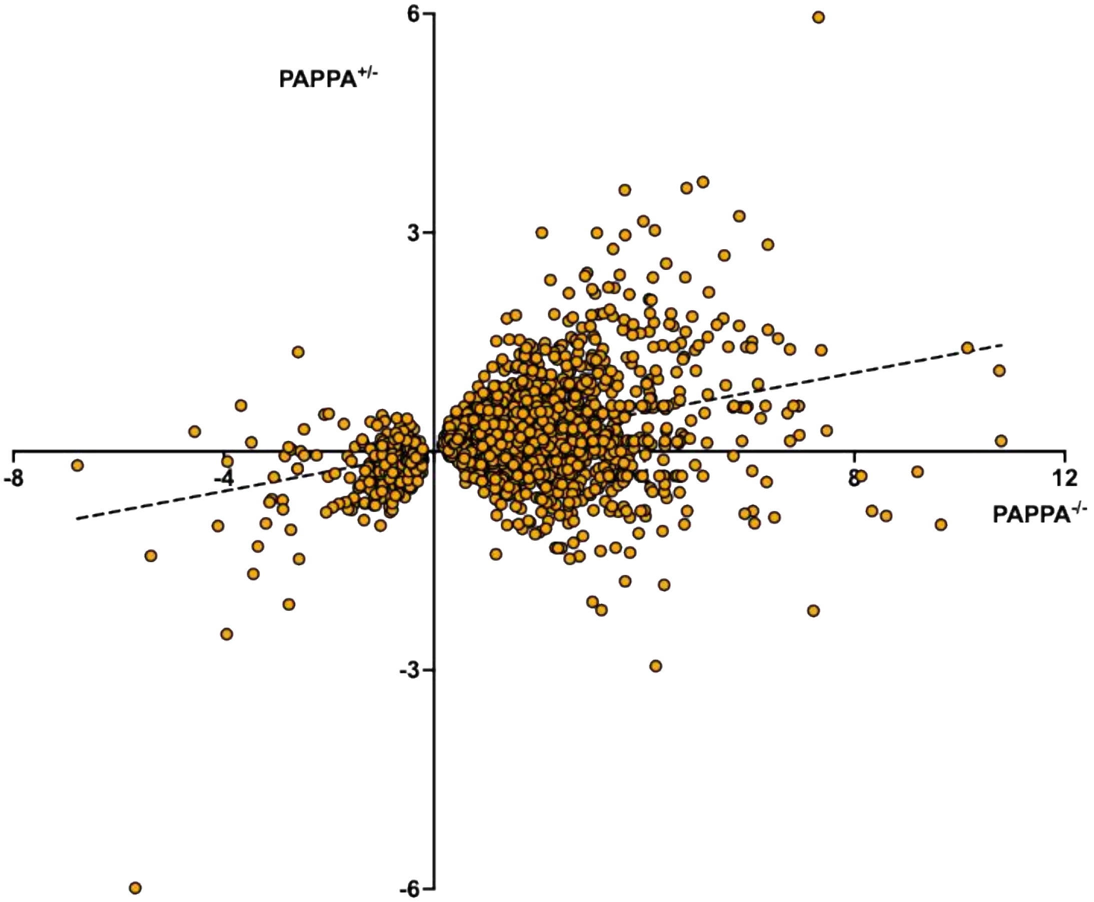

Figure S4| Gene expression in *Pappa*$^{+/-}$ vs *Pappa*$^{-/-}$ mice. Deletion of one copy of the *Pappa* gene shows a dose-dependent impact on gene expression compared to full deletion of *Pappa.* Genes that are upregulated in *Pappa*$^{-/-}$ mice tend to be upregulated in *Pappa*$^{+/-}$ mice as well. Vice versa, genes that are downregulated in *Pappa*$^{-/-}$ mice tend to be downregulated in *Pappa*$^{+/-}$ mice as well. However, the log2 fold change for each gene tends to be higher in *Pappa*$^{-/-}$ mice vs *Pappa*$^{+/-}$ mice. Depicted are all genes that are significantly up or downregulated in *Pappa*$^{-/-}$ mice. Both axes depict log2 fold changes, n = 6/group.

bioRxiv preprint doi: https://doi.org/10.1101/2025.06.03.656903; this version posted June 6, 2025. The copyright holder for this preprint (which was not certified by peer review) is the author/funder, who has granted bioRxiv a license to display the preprint in perpetuity. It is made available under aCC-BY 4.0 International license.

Figure S5

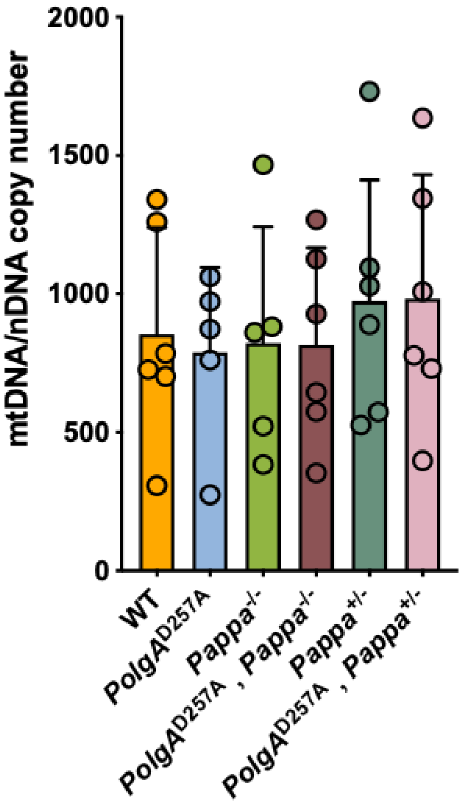

Figure S5| mtDNA copy number by qPCR in 12-month-old WT and mutant mice. qPCR detected no difference in mtDNA copy number between WT and mutant mice. N = 5-6/group.

bioRxiv preprint doi: https://doi.org/10.1101/2025.06.03.656903; this version posted June 6, 2025. The copyright holder for this preprint (which was not certified by peer review) is the author/funder, who has granted bioRxiv a license to display the preprint in perpetuity. It is made available under aCC-BY 4.0 International license.

Figure S6

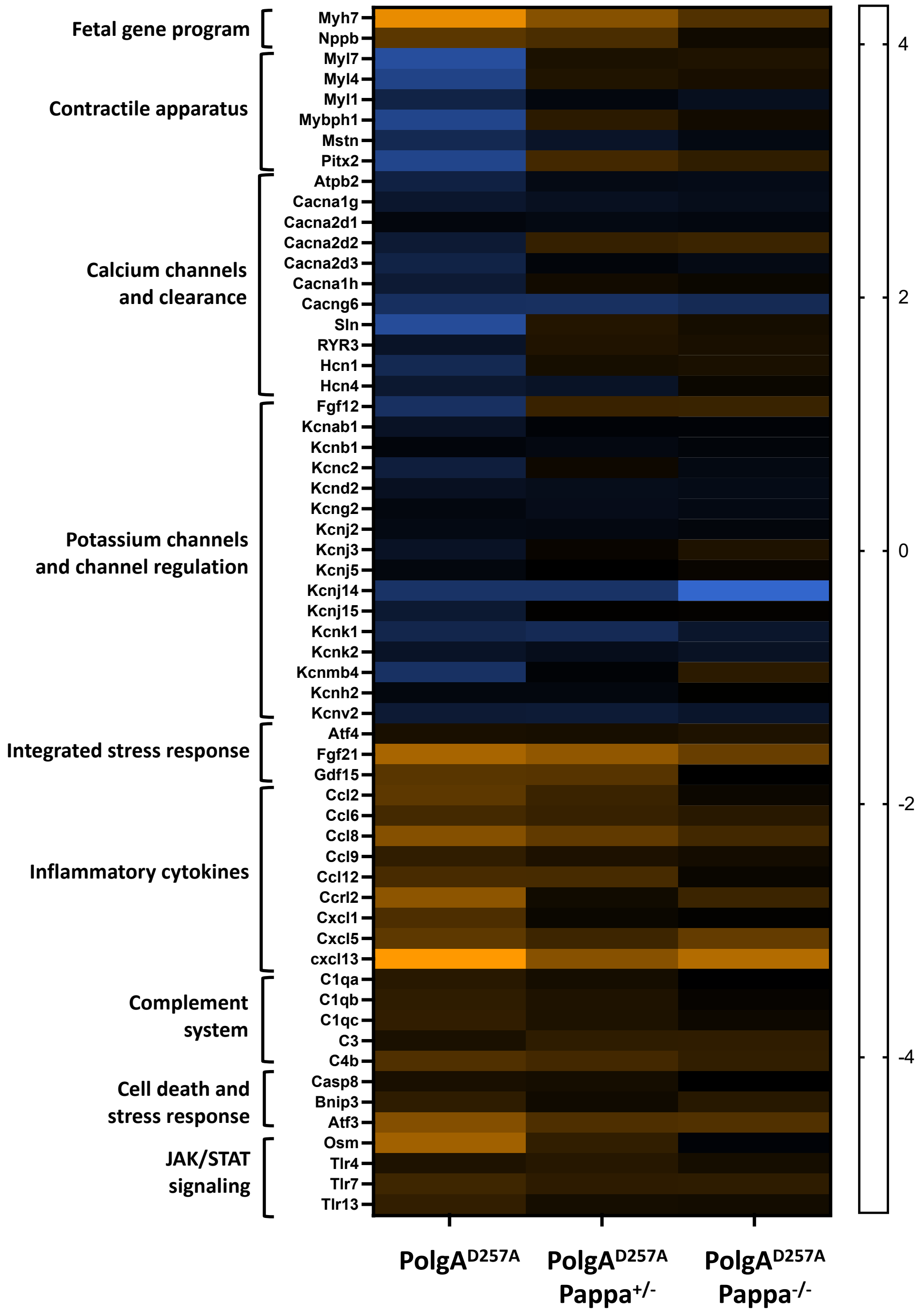

Figure S6| Expression of genes indicative of cardiomyopathy in male *PolgA*^{D257A} mice. Compared to WT mice, *PolgA*^{D257A} mice display multiple markers of cardiac stress, including increased expression of *Gdf15*, *Fgf21* and *Nppb*, as well as a reduction in the expression of genes key to contraction (*My1, Myl4 and Myl7*) and electrical signaling (calcium and potassium channels). Many of these genes move closer to WT levels upon deletion of one or two copies of the *Pappa* gene. *PolgA*^{D257A} mice also display increased levels of sterile inflammation, including inflammatory cytokines, components of the complement system, JAK/STAT signaling and markers for cell death. These inflammatory markers also seemingly subside upon deletion and depletion of the *Pappa* gene. N = 6/group.

bioRxiv preprint doi: https://doi.org/10.1101/2025.06.03.656903; this version posted June 6, 2025. The copyright holder for this preprint (which was not certified by peer review) is the author/funder, who has granted bioRxiv a license to display the preprint in perpetuity. It is made available under aCC-BY 4.0 International license.

Figure S7

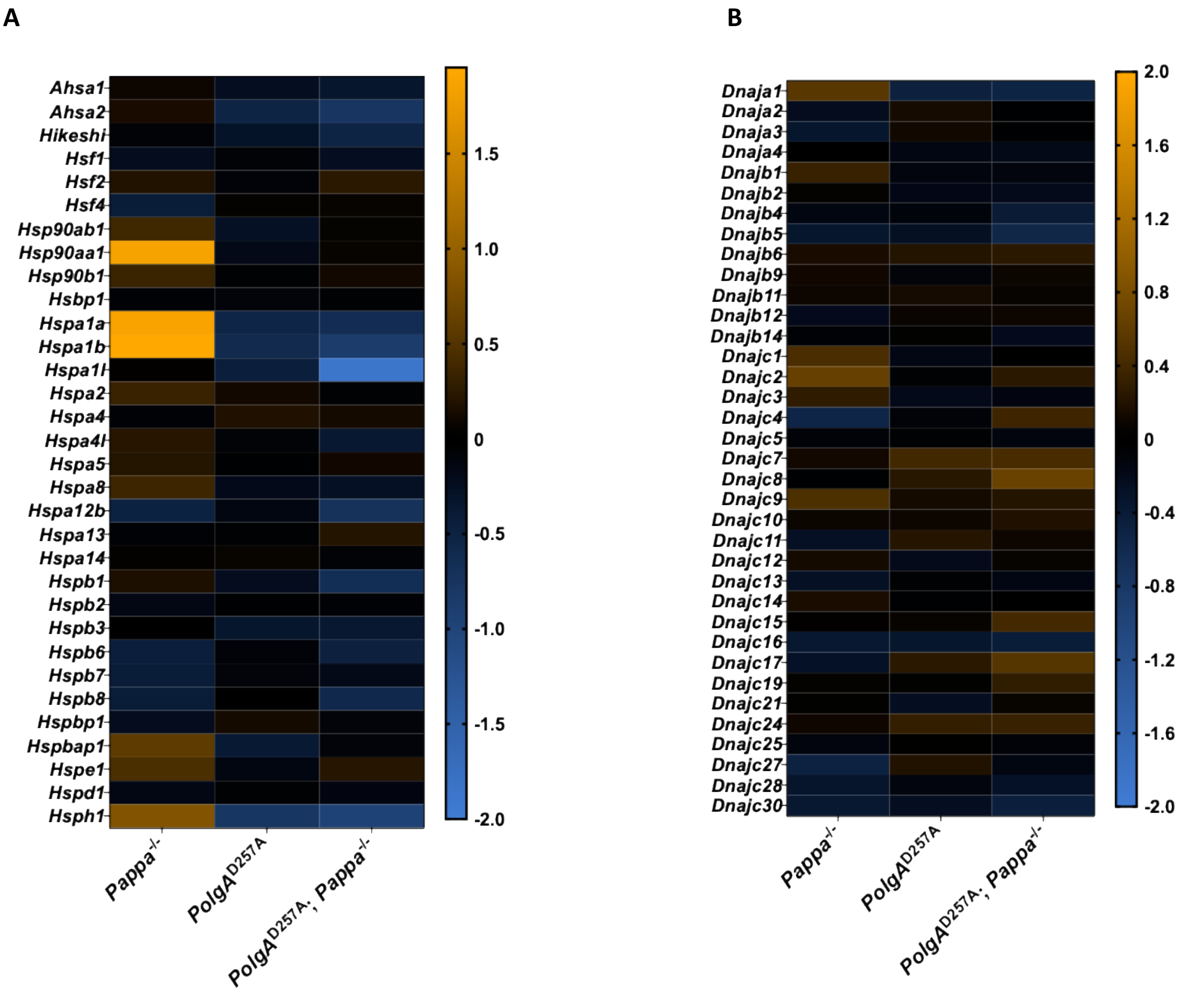

Figure S7| Expression of heat shock genes in *PolgA*^{D257A} mice with or without *Pappa* deletion. A. *PolgA*^{D257A} animals show downregulation of heat shock proteins, which is unable to be rescued by deletion of *Pappa*. These chaperones depend on ATP to function properly, stressing the importance of ATP production in their regulation. **B.** This is made clear by the loss of this trend observed in ATP-independent chaperone transcripts.
